# Efficacy of Ceragenins in Controlling the Growth of Oral Microorganisms: Implications for Oral Hygiene Management

**DOI:** 10.3390/ph17020204

**Published:** 2024-02-05

**Authors:** Michał Czarnowski, Monika Słowińska, Mariusz Sawieljew, Urszula Wnorowska, Tamara Daniluk, Grzegorz Król, Maciej Karasiński, Sławomir Okła, Paul B. Savage, Ewelina Piktel, Robert Bucki

**Affiliations:** 1 Department of Medical Microbiology and Nanobiomedical Engineering, Medical University of Białystok, 15-222 Białystok, Poland; flaps.czarnowski@gmail.com (M.C.); monika.slowinska@umb.edu.pl (M.S.); mariusz.sawieljew@umb.edu.pl (M.S.); urszula.wnorowska@umb.edu.pl (U.W.); tamara.daniluk@umb.edu.pl (T.D.); maciek.karasinski@gmail.com (M.K.); 2 Institute of Medical Science, Collegium Medicum, Jan Kochanowski University of Kielce, 25-317 Kielce, Poland; g.krol@op.pl (G.K.); slawomir.okla@gmail.com (S.O.); 3 Holy Cross Cancer Center, 25-734 Kielce, Poland; 4 Department of Chemistry and Biochemistry, Brigham Young University, Provo, UT 84602, USA; pbsavage@chem.byu.edu; 5 Independent Laboratory of Nanomedicine, Medical University of Białystok, 15-222 Białystok, Poland; ewelina.piktel@umb.edu.pl

**Keywords:** oral mouthwashes, ceragenins, oral hygiene, biofilm, antimicrobial activities, hospital-acquired infections, critically-ill patients

## Abstract

Ensuring proper dental hygiene is of paramount importance for individuals’ general well-being, particularly for patients receiving medical care. There is a prevailing utilization of conventional oral hygiene items, including toothbrushes and mouthwashes, which have gained widespread acceptance; nevertheless, their limitations encourage investigating novel options in this domain. Our study indicates that ceragenins (CSAs) being lipid analogs of host defense peptides, well-recognized for their wide-ranging antimicrobial properties, may be a potentially efficacious means to augment oral hygiene in hospitalized individuals. We demonstrate that ceragenins CSA-13, CSA-44, and CSA-131 as well as undescribed to date CSA-255 display potent antimicrobial activities against isolates of fungi, aerobic, and anaerobic bacteria from *Candida*, *Streptococcus*, *Enterococcus*, and *Bacteroides* species, which are well-recognized representatives of microbes found in the oral cavity. These effects were further confirmed against mono- and dual-species fungal and bacterial biofilms. While the ceragenins showed similar or slightly diminished efficacy compared to commercially available mouthwashes, they demonstrated a highly favorable toxicity profile toward host cells, that may translate into better maintenance of host mucosal membrane stability. This suggests that incorporating ceragenins into oral hygiene products could be a valuable strategy for reducing the risk of both oral cavity-localized and secondary systemic infections and for improving the overall health outcomes of individuals receiving medical treatment.

## 1. Introduction

Despite the notable progress achieved in medical technology and treatment modalities, bacterial infections, including those acquired in hospital environments, remain a major burden, and a common source of the organisms causing these infections are carried in the oral cavity [1]. The importance of this matter is particularly pronounced in the provision of healthcare to patients in intensive care units (ICUs) and critical care units (CCUs) [2] due to possible penetration into the circulatory system of pathogenic microorganisms through compromised mucosal barriers. For this reason, the maintenance of oral hygiene, commonly perceived as a mundane and frequently disregarded component of healthcare, should be considered crucial in the holistic welfare of those suffering from critical and life-threatening medical conditions [3].

Hospital-acquired infections (HAIs) pose a substantial risk to patients in ICUs and CCUs, resulting in extended hospitalization periods and heightened morbidity rates [4]. Since the mouth cavity harbors a diverse microbial community, constituting the second largest microbiota within the human body and consisting of various microorganisms such as bacteria, fungi, viruses, and protozoa [5], it is increasingly recognized that microbes occupying the mouth may give rise to systemic diseases if proper hygiene is not maintained [6]. For example, in a double-blind placebo-controlled multicenter study, the use of antiseptic decontamination on gingival and dental plaque resulted in a significant reduction in the colonization by aerobic bacteria in the oropharynx of patients receiving mechanical ventilation [6]. The importance of ensuring sufficient oral hygiene has been acknowledged in guideline interventions aimed at preventing ventilator-associated pneumonia (VAP) [7,8]. Based on some studies, it might be concluded that infiltration of *Streptococcus mutans*, as well as *S. sanguis*, *Staphylococcus aureus*, and *Enterococcus faecalis* is a contributing component in the progression of infective endocarditis [9,10] and infective endocarditis-related bacteremia [11,12]. Gram-negative oral bacteria and the subsequent local inflammatory response linked to periodontitis have been also identified as potential contributors to the development of chronic inflammatory disorders including respiratory diseases [13] and cardiovascular disease [14].

In ICUs, various oral care techniques have been implemented for intubated patients, including toothbrushing, proper oral suctioning, positioning, and medication adjustments as well as the application of antiseptic mouthwashes with chlorhexidine as the most widely reported antiseptic agent in the ICU [2,15]. Nevertheless, the optimal approach for ensuring adequate dental care remains uncertain, and a consensus on this matter is currently lacking [16], encouraging studies on alternative approaches for effective oral care in hospital environments.

Ceragenins (cationic antimicrobial steroids, CSAs), lipid analogs of antimicrobial peptides, are presently tested as potent and broad-spectrum antimicrobial molecules. Although ceragenins are cholic acid-based compounds, they mimic the physicochemical properties and mechanism of action of host defense peptides, particularly in terms of their membrane mechanism of action [17]. Currently, there are a number of reports demonstrating the therapeutic efficacy of this group of molecules against bacterial, fungal, and viral pathogens [18,19,20]. The investigation into the efficacy of ceragenins against oral cavity-occurring bacteria has also shown promising findings. Ceragenin CSA-13, a lipid analog of endogenous antimicrobial peptides with potent membrane-permeabilizing properties, has been shown to impede the growth of key cariogenic and periodontal pathogens, including *Porphyromonas* spp. and *S. mutans* [21]. Furthermore, these ceragenins have demonstrated efficacy in diminishing bacterial populations within biofilms [22], suggesting their capacity to disrupt established microbial communities in the oral cavity. Nevertheless, no studies have been reported demonstrating the efficacy of ceragenins against biofilms formed by oral cavity-habitating fungi and bacteria, both aerobic and anaerobic. Those pathogens are widely recognized as hospital-acquired pneumonia (HAP)-associated, particularly in immunocompromised patients or those undergoing chemotherapy and radiation therapy [23].

In this study, we present ceragenins as potential antimicrobials for the eradication of biofilm-embedded microbes in the oral cavity and their possible use in the improvement of oral hygiene management in ICU patients. The broad-spectrum activity of ceragenins covering both single- and dual-species biofilms and reduced cytotoxicity (relative to conventional mouthwashes) against host cells, including gingival fibroblasts, make them attractive candidates for further development. As research advances, ceragenins may play a pivotal role in improving the management and prevention of oral microbiota-associated diseases, ultimately contributing to better clinical outcomes in hospitalized patients.

## 2. Results

### 2.1. Potent Antimicrobial Activity of Ceragenins against All Tested Fungal and Bacterial Microorganisms Commonly Found in the Oral Cavity

In the course of the research, four ceragenins—CSA-13, CSA-44, CSA-131, and CSA-255—were tested and their activity was compared with (i) human cathelicidin-derived peptide LL-37 with well-documented broad-spectrum antimicrobial activity [24] and (ii) omiganan, an antimicrobial peptide analog of indolicidin displaying killing effects against microbes and clinically tested for treatment of skin diseases [25]. As demonstrated in Table 1 and Table 2, tested ceragenins display potent antimicrobial activity against both fungal and bacterial pathogens with MIC (minimal inhibitory concentration) values starting from 0.25 µg/mL. Particularly, CSA-13 and CSA-131 were identified as those with the most antimicrobial effects, since concentrations required to inhibit the growth of fungal and bacterial isolates ranged from 0.25 to 4 µg/mL (for CSA-13) and from 2 to 16 µg/mL (for CSA-131). Although, by comparison, CSA-255 exerted less intensified effects, its MIC values against fungal strains ranged from 4 to 8 µg/mL. The weakest antimicrobial activity among ceragenin was noted for CSA-44; nevertheless, it should be noted that its effects are still stronger than those observed for LL-37 peptide (MIC > 128 µg/mL, with the exception of anaerobic bacteria for which MIC ranges from 4 to >128 µg/mL) and omiganan (MIC range from 32 to >128 µg/mL).

**Figure 1:**
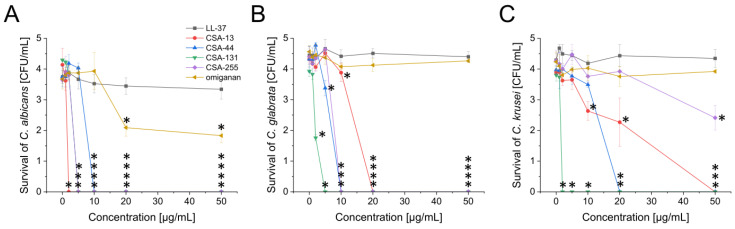
Fungicidal activity of ceragenins against *Candida* isolates from the oral cavity. Activities of CSA-13 (red circle), CSA-44 (blue triangle), CSA-131 (green inverted triangle), and CSA-255 (violet diamond) as well as LL-37 (black square) and omiganan (golden arrow) peptides were determined against representative strains of *C. albicans* (panel (**A**)), *C. glabrata* (panel (**B**)) and *C. krusei* (panel (**C**)) isolated from oral cavity using colony assay. Results are mean ± SD. * indicates statistical significance when compared to untreated control (*p* < 0.05).

Relevantly, consistent effectiveness of ceragenins was seen in these isolates, irrespective of the specific mechanism of drug resistance observed in specific isolates. As evidenced with different strains of *E. faecalis*, MIC values for CSA-13 and CSA-131 are comparable despite the different susceptibility of these isolates to vancomycin or aminoglycosides. Similarly, the resistance of *C. albicans* isolates to voriconazole does not affect the ceragenins’ activity; those isolates are characterized by low MIC values, i.e., 2 and 4 µg/mL for CSA-13 and CSA-131, respectively. To confirm the observed tendency, a colony counting assay was performed using representative fungal strains from *C. albicans*, *C. glabrata*, and *C. krusei* species. In accordance with MIC/MFC(MBC) measurements, CSA-13 and CSA-131 exhibited the most antimicrobial effects, while omiganan and LL-37 peptides were not able to eradicate tested microbes up to doses of 50 µg/mL (Figure 1).

### 2.2. Inhibition of the Formation of Mono- and Dual-Species Fungal and Bacterial Biofilms by Ceragenins

Biofilms composed of both fungal and bacterial species have the ability to adhere to various surfaces within the oral cavity, such as the teeth, gums, and mucosa, functioning as a reservoir for systemic infection-causing microbes, thereby augmenting the possibility of infection at other sites. Particularly, *Candida* biofilms have been found to increase the prevalence of ventilator-associated pneumonia due to the possibility of fungi being aspirated into the lower respiratory tract [26]. 

**Figure 2:**
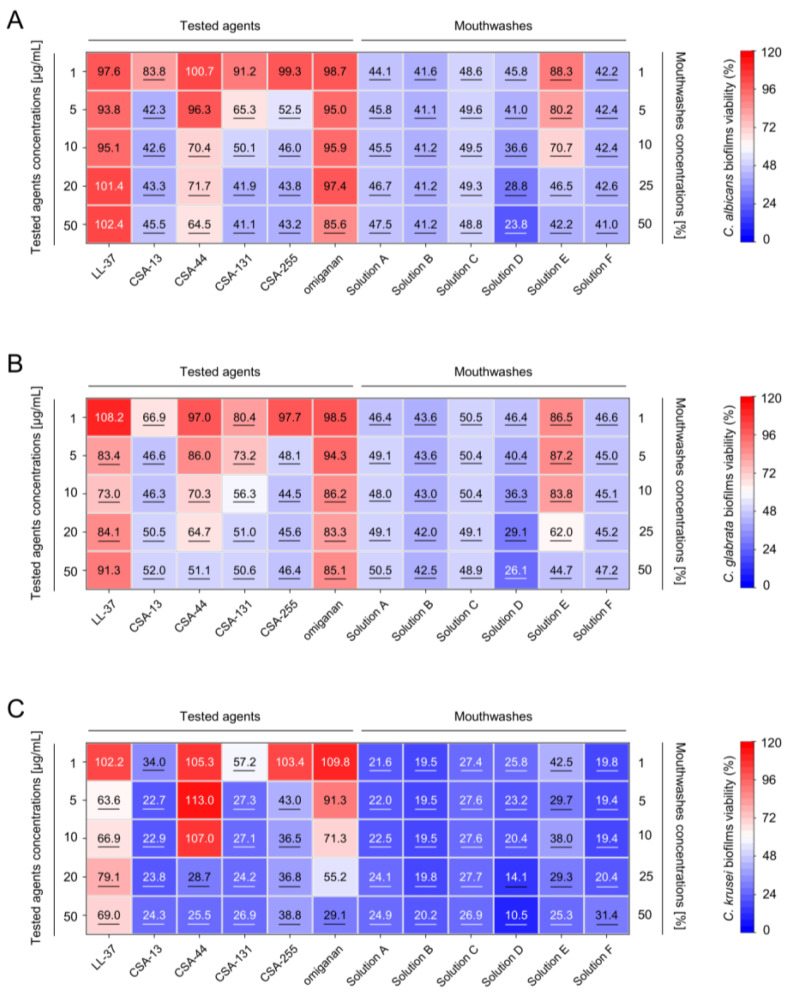
Prevention of fungal biofilm formation by tested ceragenins and peptides when compared to commercially available mouth disinfectants. Decreased formation of *C. albicans* (n = 12; panel (**A**)), *C. glabrata* (n = 7; panel (**B**)), and *C. krusei* (n = 3; panel (**C**)) biofilms upon exposure to LL-37, CSA-13, CSA-44, CSA-131 and CSA-255, omiganan (at doses ranging from 1 to 50 µg/mL) and mouthwashes (at concentrations from 1 to 50%) is presented. The results are presented as mean from all replicates for group of strains, when compared to untreated control samples, using heatmap plot. Underlined values indicate statistically significant results when compared to untreated control samples (*p* < 0.05).

We estimated the ability of tested ceragenins to inhibit the formation of biofilms formed by *C. albicans*, *C. glabrata*, and *C. krusei* laboratory and clinical isolates. As demonstrated in Figure 2, all tested ceragenins display considerable anti-biofilm activity against established, 72-h fungal biofilms as a decrease in biofilm formation at concentrations of 50 µg/mL was noted up to 58.9%, 53.6%, and 75.7% for *C. albicans*, *C. glabrata* and *C. krusei*, respectively. When comparing ceragenin-treated samples, CSA-44 exerted the least inhibitory effect. Biofilms formed by *C. krusei* were the most susceptible to ceragenin treatment. In contrast, LL-37 peptide and omiganan exhibited unsatisfactory efficiency against fungal biofilm since the inhibition rate for these agents at the highest concentration tested was not higher than ~20% for *C. albicans* and *C. glabrata*.

**Figure 3:**
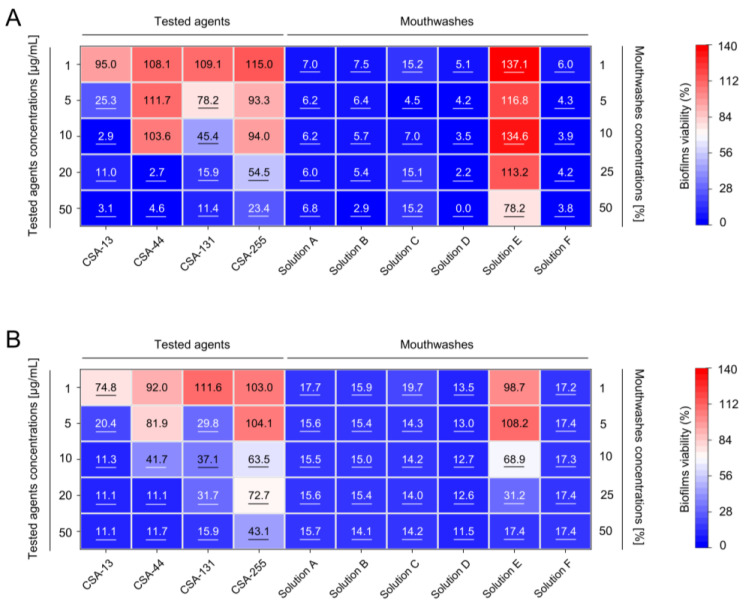
Prevention of bacterial and mixed biofilms by tested ceragenins and peptides when compared to commercially available mouth disinfectants. Decreased formation of biofilms of anaerobic bacteria (panel (**A**)) and dual-species biofilms (panel (**B**)) upon exposure to CSA-13, CSA-44, CSA-131, and CSA-255 (at doses ranging from 1 to 50 µg/mL) and mouthwashes (at concentrations from 1 to 50%) is presented. The results are presented as mean from all replicates for group of strains, when compared to untreated control samples, using heatmap plot. Underlined values indicate statistically significant results when compared to untreated control samples (*p* < 0.05).

Importantly, ceragenins exhibited potent anti-biofilm activity against biofilms formed by oral cavity-occurring bacteria, i.e., *Enterococcus faecalis* and *Streptococcus mutans*, both recognized as playing a crucial role in the development of dental plaque and thus, dental caries and periodontal diseases as well as contributing to the development of healthcare-associated infections such as ventilator-associated pneumonia [27]. According to the data presented in Figure 3A, the formation of bacterial biofilms is inhibited when exposed to ceragenin concentrations ranging from 10–20 µg/mL. Notably, CSA-13 has outstanding efficacy in reducing biofilm viability by almost 75% at a dose of 5 µg/mL. This tendency was replicated when the formation of dual-species biofilms upon ceragenin-mediated treatment was analyzed (Figure 3B). For CSA-13 and CSA-131, concentrations of 5 µg/mL were sufficient to limit the biofilm viability to 20.4% and 29.8%, respectively. Interestingly, in contrast to biofilms developed by *Candida* isolates, CSA-44 was more active than CSA-255 (no more than ~10% of viable biofilm was recorded upon exposure to 20 µg/mL of CSA-44). Overall, CSA-13 and CSA-131 exhibited comparable or slightly weaker activity than commercial oral washes. Bacterial and dual-species biofilms were more susceptible to both ceragenin- and commercial pharmaceutic-mediated inhibition of biofilm formation.

### 2.3. Low Toxicity of Ceragenins against Host Cells

**Figure 4:**
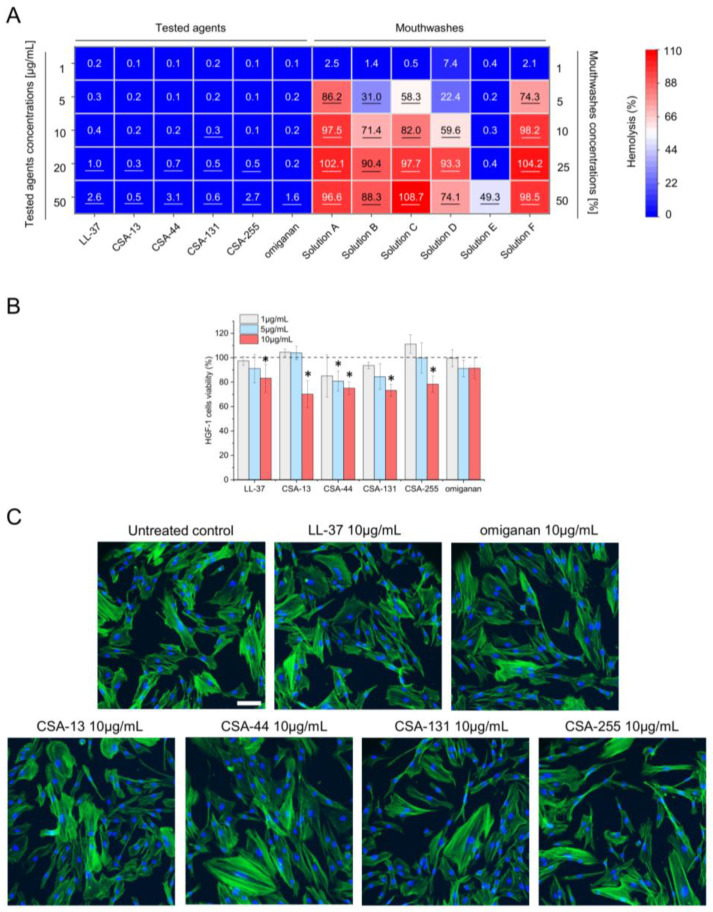
Biocompatibility of tested ceragenins in relation to host cells. Hemoglobin release from isolated erythrocytes and viability of HGF-1 (ATCC CRL-2014™) cells upon exposure to LL-37, CSA-13, CSA-44, CSA-131, and CSA-255, omiganan (at doses ranging from 1 to 50 µg/mL) and mouthwashes (at concentrations from 1 to 50%) is presented on panels (**A**,**B**), respectively. Morphology HGF-1 (ATCC CRL-2014™) subjected to compounds is presented in panel (**C**). For panels (**A**,**B**), the results from four measurements are shown. For panel (**C**), results from one representative experiment are shown. Scale ~100 µm. Underlined values (panel (**A**)) and * (panel (**B**)) indicate statistically significant results when compared to untreated control samples (*p* < 0.05).

Different oral disinfectants are widely used in clinical practice to maintain oral hygiene and prevent dental diseases. However, there has been a growing concern over the toxicity of oral mouthwashes and their impact on mucosal tissues, especially hazards linked to prolonged usage. The barrier function of the mucosal tissue in the mouth protects other tissues from the commensal flora of the oral cavity, and loss of this barrier function may lead to infection. For these reasons, it is important to ensure that newly developed oral hygienics have low toxicity to host tissues to guarantee their safe and effective use. A series of experiments was conducted with human erythrocytes and human gingival fibroblasts HGF-1 (ATCC CRL-2014™) for in vitro evaluation of the impacts of oral disinfectants on these cell types (Figure 4).

Initially, the ability of tested agents to disrupt erythrocyte membranes was determined. As demonstrated in Figure 4A, all tested peptides and ceragenins are characterized by nearly non-detectable hemolysis, as the level of released hemoglobin did not exceed 4%. In contrast, commercial mouthwashes (with the exception of Solution E) were strongly hemolytic even when diluted 10-fold (hemolysis ranging from 59.96% to 98.2%). The viability of human gingival fibroblasts HGF-1 (ATCC CRL-2014™) ranged from 91.52% to 70.16% for omiganan and CSA-13 at 10 µg/mL (Figure 4B), respectively, demonstrating high biocompatibility of ceragenins and control peptides, according to applicable standards [28]. Notably, the morphology of treated human fibroblasts was not considerably altered upon exposure of cells to 10 µg/mL of tested ceragenins and peptides (Figure 4C).

## 3. Discussion

Despite the fact that proper oral hygiene is a crucial component of overall health, it is frequently neglected in patients who are in severe conditions, both outpatients and those treated in hospitals [29,30]. Particularly, patients in critical care settings are more vulnerable to infected and inflamed teeth and gums-associated medical conditions due to inadequate immune function, the use of endotracheal tubes and ventilators, and the administration of broad-spectrum antibiotics, conditions favorable for the growth of pathogenic bacteria and facilitating the translocation of infections from the oral cavity to the other areas of the body [31,32]. For the same reason, traditional means of oral care, such as teeth brushing and using a mouthwash containing chlorhexidine, might not always be enough to prevent oral infections in a population as susceptible as these [31].

The efficacy of conventional dental care approaches is frequently limited within the context of the ICU [31]. The act of performing oral hygiene procedures, such as tooth brushing, on a patient may present challenges in cases where the patient has a medical condition or is under sedation. Furthermore, even if tooth brushing was feasible, it may not be sufficient to effectively eliminate the biofilm [33]. Moreover, despite being extensively utilized, a number of substances often found in oral mouthwashes have been identified as potentially harmful [34]. The ingredients included in this category are alcohol, antibacterial agents like chlorhexidine, and certain flavoring chemicals. While these components have the potential to provide beneficial results, they can also lead to negative consequences such as irritation of oral tissues, allergic reactions, disruption of the oral microbiota, or development of antimicrobial resistance [35]. Consequently, the challenges associated with maintaining optimal oral hygiene in critically ill patients need the exploration and implementation of innovative strategies.

In our study we compared ceragenins’ anti-biofilm activity with commercially available mouthwashes, exploring the possibility of engaging CSA-containing solutions for improved oral hygiene in vulnerable patients. In one of the first studies, Isogai et al. found that the activity of CSA-13 against cariogenic and periodontopathic bacteria, including *S. mutans* (n = 23) and *Porphyromonas* species (n = 24), demonstrated that the minimal inhibitory concentrations (MICs) of CSA-13 against the tested isolates vary between 1 and 16 µg/mL [21]. In another study, CSA-13, CSA-90, and CSA-92 were able to inhibit the growth of a spectrum of pathogens associated with oral infections (including *S. sanguinis*, *S. salivarius*, *S. pyogenes*, *E. faecalis*, *P. gingivalis*, or *Fusobacterium nucleatum*) at doses starting from 0.7 µg/mL, confirming not only potent but also broad-spectrum activity of these compounds [36]. Notably, these findings are consistent with our own results, as the MIC values of ceragenins, particularly CSA-13 and CSA-131, obtained for the fungal and bacterial strains utilized in our study fell within a similar range. Moreover, their activity was significantly greater than that recorded for the LL-37 peptide (Table 1 and Table 2), which is susceptible to inactivation by proteases [24] produced e.g., by *C. albicans* [37] or *B. fragilis* [38]. The ceragenin activities were consistent with the reports of the potent membrane-permeabilizing properties of ceragenins [17,22]. Notably, our study demonstrates the activity of ceragenins against a limited range of oral cavity microbes, as oral microbiota includes also protozoa, viruses, and non-*Candida* fungi [5]. While our research did not specifically test these microorganisms, previous findings on the activities of ceragenins [17] indicate that they will be highly effective against protozoa and viruses as well.

When comparing the anti-biofilm activities of ceragenins with commercially available mouthwashes, we recorded comparable or slightly weaker effects of ceragenin-containing solutions. However, existing literature encouraged recognition of ceragenins as antibiotics more than disinfectants as is established for oral products. Particularly, a study performed by Leszczyńska et al. revealed that some commensal strains of bacteria might be less susceptible to ceragenins than pathogenic organisms, as evidenced by *Lactobacillus casei* ssp. *casei* [36]. In another report, CSA-13 was reported to reduce the abundance of *C. difficile* in fecal samples of mice, while increasing the abundance of *Peptostreptococcaceae* bacteria as well as *Akkermansia* and *Lactobacillus* genera, demonstrating CSA-13-mediated alterations of intestinal microbiota [19]. This suggests that ceragenins might eradicate pathogenic microbes while sparing those beneficial ones, nevertheless thorough studies are required in this manner. In contrast, oral mouthwashes, containing mostly antiseptics, alcohol, and essential oils with antimicrobial activities are non-selective, and while effective, are noted to substantially alter the oral microbiome. Most recently, Liu et al. demonstrated that upon exposure to chlorhexidine and commercial mouthwashes, the abundance of bacteria from *Rothia* and *Prevotella* genera is altered, likely moving the oral microbiota toward the profiles in pneumonia patients [39]. Alterations in the oral microbiome and shifting of the oral environment toward acidic pH were also reported by Bescos et al. [35]. While the authors agreed that the impact of chlorhexidine on local and secondary diseases via altering oral microbes needs further investigation [35,39], such reports strongly suggest that non-selective mechanisms of oral rinses might be less beneficial than ceragenins for patients with treatment- or disease-associated oral dysbiosis. 

Components of saliva display both pro-biofilm and antimicrobial activities. Throughout the process of biofilm development, pathogens utilize endogenous constituents found in saliva to generate an extracellular matrix, which facilitates their co-adhesion [40], antimicrobial peptides present in saliva, such as α- and β-defensins, LL-37 peptide, lysozyme, secretory leukocyte protease inhibitor, and histatins, limiting the excessive growth of microbial populations in the human oral cavity [41]. Compelling evidence demonstrates that human saliva’s components such as mucins can compromise the biocidal activity of endogenous peptides and exogenous disinfectants used in the oral cavity [41,42,43]. Some reports suggest that the antimicrobial activity of chlorhexidine, one of the most clinically-used antiseptics (and a main component of two oral hygiene pharmaceutics tested in this study), can be diminished in the presence of saliva [42,43], and although this effect might be counteracted by the addition of 7% ethanol, its incorporation into long-term use formulation has generated considerable controversy [44]. 

A favorable feature of ceragenins is their uncompromised activity in human saliva. Our earlier study demonstrated that while antibacterial activities of LL-37 and its synthetic analog-WLBU2 are inhibited by salivary mucin, CSA-13 activity against *P. aeruginosa* PA01 and *E. coli* MG1655 are undisturbed [41]. Biofilm formation of *C. albicans* and *E. faecalis* on teeth and dental composite was also significantly inhibited despite the presence of saliva [45]. Accordingly, the presence of saliva did not affect the bactericidal and fungicidal effects of ceragenins [41,46] and was only marginally affected by the addition of dental plaque suspension [46]. These findings strongly suggest that a ceragenin-based oral formulation would not only be effective in the eradication of pathogenic microflora from the oral cavity but also would not be affected by body fluids, increasing their clinical usefulness.

The clinical applicability of ceragenin-containing oral washes is augmented by the low rate of ceragenin resistance induction in pathogenic bacteria and fungi. This is especially important in light of the multiple reports revealing lowered susceptibility of some multidrug-resistant Gram-positive and Gram-negative bacterial isolates, including pan-resistant *Acinetobacter* and *Klebsiella* or methicillin-resistant *S. aureus* (MRSA), to elevated (2%) concentrations of chlorhexidine upon prolonged exposure to this antiseptic [47]. It has been also observed that chlorhexidine induces VanA-type vancomycin resistance genes in *Enterococcus* species [48] and upregulates the proteins involved in the assembly of lipopolysaccharide (LPS) for outer membrane biogenesis and virulence factors in *P. aeruginosa* [49]. Moreover, the findings of some clinical trials have sparked concerns regarding the possibility of selection of hospital-acquired infections, reduced susceptibility to chlorhexidine, and the development of cross-resistance to clinically utilized antibiotics [50]. Indeed, it was confirmed that chlorhexidine-resistant organisms exhibited reduced susceptibility to colistin, even in the absence of prior exposure to colistin [49]. At the same time, it was confirmed that the usage of chlorhexidine does not alter the susceptibility of these strains to ceragenins [49]. Reports on the induction of microbial drug resistance by ceragenins are even more promising; reports on this topic highlight the inability of bacteria to develop resistance to these antimicrobials. In a study by Leszczyńska et al., *S. aureus* cells were not able to develop adaptive survival strategies upon exposure to CSA-13 [36]. Among five *Candida* clinical isolates tested by Spałek et al., none was able to develop resistance to CSA-131 (MICs not higher than 2 µg/mL for 25 passages), although some increase in MICs of CSA-13 were noted [51]. In another study, CSA-13 retained its bactericidal activity against *S. aureus* for 30 passages but induced some insensitivity in Gram-negative isolates upon several passages [52]. 

The development of oral mouthwash products with minimal toxicity is of utmost importance in order to facilitate their effective utilization in clinical settings. To date, some reports suggested the potential harmfulness and an increase in oral cancer development due to mouthwashes [53,54], particularly those that contain alcohol [34] and are used over the long term; nevertheless, further meta-analyses did not confirm these conclusions [44,55]. Regardless, due to the regular and extended usage of oral hygiene products, it is crucial to conduct constant studies and monitoring of the oral toxicity associated with mouthwashes, and it is important to continually improve their formulation and minimize any potential risks associated with their use. 

In this study, we demonstrate that tested ceragenins do not exert toxic effects against mammalian cells at doses that are antimicrobial and effective against established biofilms. In addition, hemolysis was at non-detectable levels up to 50 µg/mL with a majority of the compounds. At 10 µg/mL cytotoxicity against human gingival cells did not exceed 30%, which, according to the International Standard ISO 10993-5:2009(E) [28], indicates appropriate biocompatibility of tested chemical. At the same time, tested commercial mouthwashes displayed considerably more toxic effects both when exploring the release of hemoglobin from damaged erythrocytes and when analyzing the viability of gingival fibroblasts. Notably, both erythrocytes and HGF-1 (ATCC CRL-2014™) cells were exposed to diluted oral products and ceragenins for a prolonged time (1 h and 24 h, respectively) so this effect might not be as severe in in vivo settings; nevertheless, there is a clear and distinct difference in cytotoxic effects between the currently available pharmaceutics and ceragenin-containing formulations. This observation provides solid evidence to support the potential use of ceragenin-based mouthwashes in oral care scenarios, including everyday oral hygiene and particularly for patients after surgical procedures or those experiencing hypersensitivity resulting from oral mucosa damage and local inflammation. Particularly, the group of cancer patients, including those head and neck cancer patients receiving radiation therapy or chemotherapy and thus, suffering from oral mucositis [56], might strongly benefit from these features of ceragenins. 

Presently, the utilization of mouthwashes that incorporate corticosteroids and nonsteroidal anti-inflammatory medications has proven to be efficacious in the prevention or mitigation of mucositis linked to cancer treatment [57,58]. Direct and indirect anti-inflammatory features have been noted in some mouthwashes and toothpaste containing compounds such as chlorhexidine, cetylpyridinium chloride, or stannous fluoride, which are utilized in controlling gingivitis, supragingival plaque formation, and periodontal disease development [59]. The existing literature on the anti-inflammatory and regenerative activities of ceragenins [22,60] implies that ceragenins may have potential applications in a comparable context. Anti-inflammatory properties of CSA-13 were confirmed in animal models of *P. aeruginosa*-induced peritonitis [60] and *E. coli*-associated urinary tract infection [61]. In another study conducted by Bucki et al., it was also shown that ceragenin CSA-13 possesses the capability to inhibit cellular pro-inflammatory pathways triggered by lipopolysaccharide. Consequently, this inhibitory effect may serve to avoid the occurrence of systemic inflammation resulting from bacterial wall components [60]. These potential effects described for ceragenins may have a beneficial impact on the management of oral inflammation caused by treatment modalities or inadequate oral hygiene habits, particularly when combined with the regenerative features of ceragenins. Although, to date, no research was performed using oral cavity-isolated cells, it was demonstrated that ceragenins CSA-13 and CSA-192 are able to inhibit the formation of bacterial biofilms and simultaneously stimulate migration of human keratinocytes and promote tube formation by VEGFR2-mediated signaling pathway [22]. Ceragenin CSA-13 at concentrations up to 10 µg/mL was also recorded to enhance the proliferation of human osteoblasts [46], strongly suggesting that ceragenin-containing oral washes could be effectively utilized for individuals requiring periodontal regeneration and following surgical procedures and dental treatments.

Collectively, ceragenins possess a broad spectrum of favorable features, demonstrated both in this study and evidenced in the literature, that could make them an innovative compound for oral formulations. Particularly, their broad-spectrum antimicrobial actions targeting all clinically relevant oral pathogens, both in planktonic and biofilm form, strongly encourage their employment in the management of oral hygiene. Importantly, ceragenins were demonstrated to be efficient against antibiotic-susceptible and drug-resistant pathogens which strongly suggests that oral mouthwashes containing these agents would be highly effective in oral pathogens’ eradication. On the contrary, the antibacterial activity of commercial mouthwashes is contingent upon the primary active ingredients they contain [62]. Specifically, only mouthwash solutions comprising chlorohexidine gluconate or cetylpyridinum chloride demonstrated efficacy against the majority of bacterial strains examined in their biofilm state, although not all [63]. In this aspect, ceragenins carry the potential to improve this effect. Moreover, in our in vitro study, ceragenins were demonstrated to be less cytotoxic than commercial formulations, allowing us to hypothesize that the use of CSA-containing solutions will avoid some adverse effects of mouthwashes, including irritation, mild desquamation, or mucosal ulceration [64] and thus, will be better tolerated by patients. Moreover, ceragenins as easily water-soluble molecules [65] would not require additional transport vehicles or additional solvents, diminishing the likelihood of intolerances [66]. The uncompromised activity in human saliva, anti-inflammatory and regenerative properties of ceragenins, as well as their ability to modulate microbiome composition may be strongly beneficial in this context.

The use of ceragenins in oral formulations carries significant implications for sustainability, encompassing economic, social, and environmental aspects. Particularly, the inclusion of ceragenins into oral hygiene appears to align with sustainability principles by addressing oral health issues in a preventive manner, potentially reducing the reliance on antibiotics by the prevention of secondary systemic infections and limiting the drug resistance spread, as well as minimizing medical complications, and promoting long-term health outcomes. Implementing this proactive strategy not only enhances personal welfare but also fosters the long-term viability of healthcare systems by encouraging a more effective allocation of resources.

Our study has several identified limitations that should be considered for further analysis of the clinical application of the tested compounds. First, the study was conducted on only a limited number of strains, thus not considering the full spectrum of microorganisms that comprise the oral microbiota. In this study, we also did not perform analyses evaluating the effects of these compounds on commensal microorganisms, which should be the subject of further analyses. Our analyses were also carried out using aqueous solutions of ceragenin; however, other transport media containing additional components such as fluoride ions should be investigated to obtain the most clinically beneficial formulations. In addition, microbiological analyses should be carried out, preferably after long-term use of these substances, assessing whether there are changes in the composition of the natural oral microbiota and whether the selection of ceragenin-resistant microorganisms is observed. Since all experiments were conducted in vitro, it is imperative to do additional studies in order to comprehensively assess the safety of using ceragenins, particularly after prolonged use, both in terms of acute reactions (such as irritation) and those of a chronic nature. All these characteristics should be comprehensively explored in potential clinical trials of CSA-containing products. 

## 4. Materials and Methods

### 4.1. Material

CSA-13, CSA-44, CSA-131, and CSA-255 were synthesized as described previously [67]. Cathelicidin LL-37 and omiganan were synthetized by Lipopharm.pl (Zblewo, Poland). Reagents for colorimetric, fluorescent-based, and microscopic analyses, namely, resazurin sodium salt, Hoechst 33342, and thiazolyl blue tetrazolium bromide (MTT) were purchased from Sigma Aldrich (St. Louis, MO, USA). Fluorescent-labeled phalloidin was from ThermoFisher Scientific (Waltham, MA, USA). Culture media, including Sabouraud 4% dextrose agar, Columbia agar +5% sheep blood, Brain Heart Infusion (BHI) agar, and Tryptic Soy Broth (TSB) were obtained from BioMaxima (Lublin, Poland). The VITEK 2R Fungal Susceptibility Cards (AST-YS07) were acquired from bioMerieux (Marcy-l’Etoile, France). Commercially available mouthwashes containing chlorhexidine digluconate, cetylpyridinium chloride, octenidine, or essential oils (with or without alcohol), were used to compare the antimicrobial activity of ceragenins to conventional products.

### 4.2. Fungal and Bacterial Strains

For the purpose of the study, 22 laboratory and clinical strains of *Candida* spp., 8 strains of aerobic bacteria (including 5 strains of *E. faecalis*, 2 strains of *S. mutans*, and 1 strain of *E. hirae*), as well as 5 strains of anaerobic bacteria (including 3 strains of *Bacteroides fragilis*) were used. A total of 21 clinical strains of *Candida* fungi (11 clinical strains of *C. albicans*, 7 clinical isolates of *C. glabrata*, and 3 clinical isolates of *C. krusei*; all isolated from the oral cavity), 3 clinical isolates of *E. faecalis* and *S. mutans*, as well as 5 isolates of anaerobic bacteria were obtained from the collection of Department of Medical Microbiology and Nanobiomedical Engineering (Medical University of Bialystok, Białystok, Poland). A laboratory strain of *C. albicans* (*C. albicans* 1408) was obtained from the Polish Collection of Microorganisms, Polish Academy of Science (Wroclaw, Poland). Laboratory bacterial strains were from the American Type Culture Collection (ATCC).

### 4.3. Antimicrobial Testing

The minimal inhibitory concentration (MIC) and minimal fungicidal/bactericidal concentration (MFC/MBC) were determined using a broth microdilution method to estimate the therapeutic potential of tested peptides and ceragenins against yeast and anaerobic and aerobic bacteria cultured in high-nutritious media. Briefly, tested isolates were grown on Sabouraud 4% dextrose agar (for *Candida* spp.), Columbia agar +5% sheep blood (for *E. faecalis* and *S. mutans* strains), or BHI agar plates (for anaerobic bacterial isolates), harvested, resuspended in TSB or BHI broth and adjusted by serial dilutions to ∼2 × 10^5^ CFU/mL. The final concentrations of the tested agents ranged from 128 µg/mL to 0.25 µg/mL. MIC was visually ascertained as the lowest concentration of the tested chemical that exhibited no growth of microorganisms following an 18–24 h incubation period. The fungicidal and bactericidal activity of the samples was assessed by inoculating 10 µL of each sample onto agar plates [46]. In another setting of the experiment, the fungicidal activities of the tested agent were determined using a colony counting assay, according to the previously published protocol [46,68].

### 4.4. Biofilm Viability Measurements

The anti-biofilm efficacy of ceragenins CSA-13, CSA-44, CSA-131, and CSA-255 as well as LL-37 and omiganan peptides against biofilm-embedded pathogens, when compared to commercial mouthwashes, was assessed using resazurin-based fluorimetric staining [69,70], which facilitate the measurement of live biofilm cells cultivated in microtiter plates. Accordingly, tested fungal and bacterial isolates at the logarithmic phase of growth were suspended in TSB broth at an optical density (OD) of ~0.5 (for *Candida* spp.) or 0.1 (for bacterial strains), diluted 50-fold, distributed into 96-well flat-bottom plates, and exposed to tested ceragenins and peptides at doses ranging from 1 to 50 µg/mL for 72 h at 37 °C. Upon removing of planktonic cells, resazurin at a final concentration of 200 µg/mL was added. The assessment of biofilm viability was conducted by measuring the fluorescence intensity at excitation/emission wavelengths of 520/590 nm using a Varioskan Lux (ThermoFisher Scientific (Waltham, MA, USA) microplate reader.

### 4.5. Biocompatibility Assessment

As an initial indicator of the toxicity of ceragenins towards host cells, the disruption of red blood cell (RBC) membranes upon treatment with ceragenins was used with RBCs isolated from the blood of healthy volunteers and suspended in sterile PBS to achieve a hematocrit of 10% [71,72]. To explore the cytocompatibility towards gingival fibroblasts, a cell culture model was engaged. For this purpose, HGF-1 (ATCC CRL-2014™) cells were maintained in high-glucose DMEM (Dulbecco’s Modified Eagle Medium) supplemented with 20% fetal bovine serum (FBS), glutamine (2 mM/L), and Antibiotic Antimycotic Solution (1%) at 37 °C and in an atmosphere containing 5% CO_2_ with saturated humidity. For experiments, cells were seeded at a density of 2.6 × 10^3^ cells/well and exposed to tested agents for 24 h before the MTT assay was performed [60,73]. To visualize the morphology of treated gingival fibroblast cells, the cytoskeleton and nuclei of cells were stained and analyzed using fluorescence microscopy. For this purpose, cells treated with ceragenins/peptides for 24 h were fixed with 3.7% formaldehyde in PBS, permeabilized with ice-cold Triton X-100 (0.1% in PBS) and stained with rhodamine-labeled phalloidin (1 U/mL for 30min at room temperature) and Hoechst 33342 (1 µg/mL for 1 min at room temperature) with thorough washing of cells with PBS between each step [60].

### 4.6. Statistical Analysis

Statistical significance was determined using a One-way ANOVA test and performed using OriginLab 2023b software.

## 5. Conclusions

Our study demonstrated that ceragenins might be effective as a component of oral mouthwashes, due to their broad-spectrum antimicrobial activity against both planktonic and biofilm-embedded pathogens and their low cytotoxicity against gingival fibroblasts. 

## Figures and Tables

**Table 1:** Minimal inhibitory concentrations (MIC) and minimal fungicidal concentrations (MFC), [µg/mL] of LL-37 peptide, ceragenins CSA-13, CSA-44, CSA-131, and CSA-255, and omiganan peptide against tested *Candida*.

	LL-37	CSA-13	CSA-44	CSA-131	CSA-255	Omiganan
*C. albicans 1408*	>128/>128	4/4	64/64	16/16	8/8	>128/>128
*C. albicans* *	>128/>128	4/4	32/32	16/64	16/16	128/128
*C. albicans* *	>128/>128	2/2	16/16	4/4	8/8	128/128
*C. albicans* *	>128/>128	2/2	64/64	4/4	8/8	128/128
*C. albicans* *	>128/>128	2/2	16/32	4/4	8/8	128/128
*C. albicans* *	>128/>128	2/2	16/32	2/2	8/8	128/128
*C. albicans* *	>128/>128	2/2	16/64	4/4	8/8	128/128
*C. albicans* *	>128/>128	2/16	16/32	4/4	8/8	128/128
*C. albicans* *	>128/>128	4/4	32/128	32/32	32/32	128/128
*C. albicans* *	>128/>128	2/2	64/64	8/32	4/32	32/64
*C. albicans* *	>128/>128	2/16	16/>128	4/4	8/8	128/128
*C. albicans* *	>128/>128	4/4	32/32	8/8	8/8	128/128
*C. glabrata* *	>128/>128	4/4	128/128	4/4	8/8	>128/>128
*C. glabrata* *	>128/>128	2/2	128/128	2/32	8/32	128/128
*C. glabrata* *	>128/>128	1/8	8/8	4/16	4/4	128/128
*C. glabrata* *	>128/>128	1/4	16/32	2/2	8/8	128/>128
*C. glabrata* *	>128/>128	1/1	8/8	2/2	8/8	>128/>128
*C. glabrata* *	>128/>128	1/1	16/16	128/128	8/8	>128/>128
*C. glabrata* *	>128/>128	1/8	8/64	2/2	8/64	>128/>128
*C. krusei* *	>128/>128	1/1	8/8	2/2	4/8	64/64
*C. krusei* *	>128/>128	1/1	8/8	2/4	4/4	64/64
*C. krusei* *	>128/>128	1/4	8/8	2/2	4/4	32/32

* Clinical isolate.

**Table 2:** Minimal inhibitory concentrations (MIC), and minimal bactericidal concentrations (MBC) [µg/mL] of LL-37 peptide, CSA-13, CSA-44, CSA-131, and CSA-255 against anaerobic and aerobic oral cavity infection-associated bacteria.

	LL-37	CSA-13	CSA-44	CSA-131	CSA-255
*E. faecalis ATCC 45477*	>128/>128	2/4	64/64	8/8	>128/>128
*E. faecalis ATCC 51575*	>128/>128	2/16	64/64	8/8	64/128
*E. faecalis ATCC 29212*	>128/>128	0.5/>4	64/128	4/8	64/64
*E. faecalis* *	>128/>128	2/16	64/64	4/4	128/128
*E. faecalis* *	>128/>128	0.25/2	64/64	4/8	32/128
*S. mutans ATCC 35668*	>128/>128	1/>8	64/64	16/16	64/64
*S. mutans* *	>128/>128	1/8	128/128	128/128	128/128
*E. hirae ATCC 10541*	>128/>128	0.5/>4	64/64	4/4	64/64
*B. fragilis* *	>128/>128	0.5/4	64/>128	8/64	32/>128
*B. fragilis* *	8/>64	1/1	64/64	4/8	8/16
*B. fragilis* *	4/8	1/1	64/64	16/32	16/32
*Capnocytophaga* *	8/>64	0.5/1	32/128	4/8	8/64
*Prevotella* *	8/>64	1/2	32/128	4/8	16/16

* Clinical isolate.

## Data Availability

The data presented in this study are available on request from the corresponding author.

## References

[1] Sjögren P., Wårdh I., Zimmerman M., Almståhl A., Wikström M. (2016). Oral Care and Mortality in Older Adults with Pneumonia in Hospitals or Nursing Homes: Systematic Review and Meta-Analysis. J. Am. Geriatr. Soc..

[2] Rello J., Koulenti D., Blot S., Sierra R., Diaz E., De Waele J.J., Macor A., Agbaht K., Rodriguez A. (2007). Oral care practices in intensive care units: A survey of 59 European ICUs. Intensive Care Med..

[3] Salamone K., Yacoub E., Mahoney A.M., Edward K.L. (2013). Oral care of hospitalised older patients in the acute medical setting. Nurs. Res. Pract..

[4] Blot S., Ruppé E., Harbarth S., Asehnoune K., Poulakou G., Luyt C.E., Rello J., Klompas M., Depuydt P., Eckmann C. (2022). Healthcare-associated infections in adult intensive care unit patients: Changes in epidemiology, diagnosis, prevention and contributions of new technologies. Intensive Crit. Care Nurs..

[5] Deo P.N., Deshmukh R. (2019). Oral microbiome: Unveiling the fundamentals. J. Oral. Maxillofac. Pathol..

[6] Fourrier F., Dubois D., Pronnier P., Herbecq P., Leroy O., Desmettre T., Pottier-Cau E., Boutigny H., Di Pompéo C., Durocher A. (2005). Effect of gingival and dental plaque antiseptic decontamination on nosocomial infections acquired in the intensive care unit: A double-blind placebo-controlled multicenter study. Crit. Care Med..

[7] Hellyer T.P., Ewan V., Wilson P., Simpson A.J. (2016). The Intensive Care Society recommended bundle of interventions for the prevention of ventilator-associated pneumonia. J. Intensive Care Soc..

[8] Maryani N., Octavia A., Budiyantoro C., Ulfa M. (2023). Prevention of Pneumonia due to Ventilator in Critical Patients with U Shape Oral Hygiene Model: A Systematic Review. Rom. J. Anaesth. Intensive Care.

[9] Abranches J., Zeng L., Bélanger M., Rodrigues P.H., Simpson-Haidaris P.J., Akin D., Dunn W.A., Progulske-Fox A., Burne R.A. (2009). Invasion of human coronary artery endothelial cells by Streptococcus mutans OMZ175. Oral. Microbiol. Immunol..

[10] Del Giudice C., Vaia E., Liccardo D., Marzano F., Valletta A., Spagnuolo G., Ferrara N., Rengo C., Cannavo A., Rengo G. (2021). Infective Endocarditis: A Focus on Oral Microbiota. Microorganisms.

[11] Lockhart P.B., Brennan M.T., Thornhill M., Michalowicz B.S., Noll J., Bahrani-Mougeot F.K., Sasser H.C. (2009). Poor oral hygiene as a risk factor for infective endocarditis-related bacteremia. J. Am. Dent. Assoc..

[12] Eleftheriotis G., Marangos M., Lagadinou M., Bhagani S., Assimakopoulos S.F. (2023). Oral Antibiotics for Bacteremia and Infective Endocarditis: Current Evidence and Future Perspectives. Microorganisms.

[13] Linden G.J., Lyons A., Scannapieco F.A. (2013). Periodontal systemic associations: Review of the evidence. J. Clin. Periodontol..

[14] Sanz M., Marco Del Castillo A., Jepsen S., Gonzalez-Juanatey J.R., D’Aiuto F., Bouchard P., Chapple I., Dietrich T., Gotsman I., Graziani F. (2020). Periodontitis and cardiovascular diseases: Consensus report. J. Clin. Periodontol..

[15] Miranda A.F., de Paula R.M., de Castro Piau C.G., Costa P.P., Bezerra A.C. (2016). Oral care practices for patients in Intensive Care Units: A pilot survey. Indian. J. Crit. Care Med..

[16] Torres A., Niederman M.S., Chastre J., Ewig S., Fernandez-Vandellos P., Hanberger H., Kollef M., Li Bassi G., Luna C.M., Martin-Loeches I. (2017). International ERS/ESICM/ESCMID/ALAT guidelines for the management of hospital-acquired pneumonia and ventilator-associated pneumonia: Guidelines for the management of hospital-acquired pneumonia (HAP)/ventilator-associated pneumonia (VAP) of the European Respiratory Society (ERS), European Society of Intensive Care Medicine (ESICM), European Society of Clinical Microbiology and Infectious Diseases (ESCMID) and Asociación Latinoamericana del Tórax (ALAT). Eur. Respir. J..

[17] Dias C., Rauter A.P. (2019). Membrane-targeting antibiotics: Recent developments outside the peptide space. Future Med. Chem..

[18] Neves A.R., Freitas-Silva J., Durães F., Silva E.R., Rodrigues I.C., Mergulhão F., Gomes M., Teixeira-Santos R., Bernardes André M., Silva R. (2023). Insights into the antimicrobial properties of a cationic steroid and antibiofilm performance in PDMS-based coatings to potentially treat urinary infections. J. Mater. Chem. B.

[19] Wang J., Ghali S., Xu C., Mussatto C.C., Ortiz C., Lee E.C., Tran D.H., Jacobs J.P., Lagishetty V., Faull K.F. (2018). Ceragenin CSA13 Reduces Clostridium difficile Infection in Mice by Modulating the Intestinal Microbiome and Metabolites. Gastroenterology.

[20] Sinclair K.D., Pham T.X., Farnsworth R.W., Williams D.L., Loc-Carrillo C., Horne L.A., Ingebretsen S.H., Bloebaum R.D. (2012). Development of a broad spectrum polymer-released antimicrobial coating for the prevention of resistant strain bacterial infections. J. Biomed. Mater. Res. A.

[21] Isogai E., Isogai H., Takahashi K., Okumura K., Savage P.B. (2009). Ceragenin CSA-13 exhibits antimicrobial activity against cariogenic and periodontopathic bacteria. Oral. Microbiol. Immunol..

[22] Olekson M.A., You T., Savage P.B., Leung K.P. (2017). Antimicrobial ceragenins inhibit biofilms and affect mammalian cell viability and migration. FEBS Open Bio.

[23] Rathbun K.P., Bourgault A.M., Sole M.L. (2022). Oral Microbes in Hospital-Acquired Pneumonia: Practice and Research Implications. Crit. Care Nurse.

[24] Ridyard K.E., Overhage J. (2021). The Potential of Human Peptide LL-37 as an Antimicrobial and Anti-Biofilm Agent. Antibiotics.

[25] Niemeyer-van der Kolk T., van der Wall H., Hogendoorn G.K., Rijneveld R., Luijten S., van Alewijk D.C.J.G., van den Munckhof E.H.A., de Kam M.L., Feiss G.L., Prens E.P. (2020). Pharmacodynamic Effects of Topical Omiganan in Patients With Mild to Moderate Atopic Dermatitis in a Randomized, Placebo-Controlled, Phase II Trial. Clin. Transl. Sci..

[26] Delisle M.S., Williamson D.R., Albert M., Perreault M.M., Jiang X., Day A.G., Heyland D.K. (2011). Impact of Candida species on clinical outcomes in patients with suspected ventilator-associated pneumonia. Can. Respir. J..

[27] Sands K.M., Twigg J.A., Lewis M.A.O., Wise M.P., Marchesi J.R., Smith A., Wilson M.J., Williams D.W. (2016). Microbial profiling of dental plaque from mechanically ventilated patients. J. Med. Microbiol..

[28] Biological Evaluation of Medical Devices—Part 5: Tests for In Vitro Cytotoxicity.

[29] Dagnew Z.A., Abraham I.A., Beraki G.G., Tesfamariam E.H., Mittler S., Tesfamichael Y.Z. (2020). Nurses’ attitude towards oral care and their practicing level for hospitalized patients in Orotta National Referral Hospital, Asmara-Eritrea: A cross-sectional study. BMC Nurs..

[30] Janto M., Iurcov R., Daina C.M., Neculoiu D.C., Venter A.C., Badau D., Cotovanu A., Negrau M., Suteu C.L., Sabau M. (2022). Oral Health among Elderly, Impact on Life Quality, Access of Elderly Patients to Oral Health Services and Methods to Improve Oral Health: A Narrative Review. J. Pers. Med..

[31] Kelly N., Blackwood B., Credland N., Stayt L., Causey C., Winning L., McAuley D.F., Lundy F.T., El Karim I. (2023). Oral health care in adult intensive care units: A national point prevalence study. Nurs Crit Care..

[32] Zhao T., Wu X., Zhang Q., Li C., Worthington H.V., Hua F. (2020). Oral hygiene care for critically ill patients to prevent ventilator-associated pneumonia. Cochrane Database Syst. Rev..

[33] Cvikl B., Lussi A. (2021). Supragingival Biofilm: Toothpaste and Toothbrushes. Monogr. Oral. Sci..

[34] Calderón-Montaño J.M., Jiménez-Alonso J.J., Guillén-Mancina E., Burgos-Morón E., López-Lázaro M. (2018). A 30-s exposure to ethanol 20% is cytotoxic to human keratinocytes: Possible mechanistic link between alcohol-containing mouthwashes and oral cancer. Clin. Oral. Investig..

[35] Bescos R., Ashworth A., Cutler C., Brookes Z.L., Belfield L., Rodiles A., Casas-Agustench P., Farnham G., Liddle L., Burleigh M. (2020). Effects of Chlorhexidine mouthwash on the oral microbiome. Sci. Rep..

[36] Leszczynska K., Namiot D., Byfield F.J., Cruz K., Zendzian-Piotrowska M., Fein D.E., Savage P.B., Diamond S., McCulloch C.A., Janmey P.A. (2013). Antibacterial activity of the human host defence peptide LL-37 and selected synthetic cationic lipids against bacteria associated with oral and upper respiratory tract infections. J. Antimicrob. Chemother..

[37] Rapala-Kozik M., Bochenska O., Zawrotniak M., Wolak N., Trebacz G., Gogol M., Ostrowska D., Aoki W., Ueda M., Kozik A. (2015). Inactivation of the antifungal and immunomodulatory properties of human cathelicidin LL-37 by aspartic proteases produced by the pathogenic yeast Candida albicans. Infect. Immun..

[38] Gibson S.A., Macfarlane G.T. (1988). Studies on the proteolytic activity of Bacteroides fragilis. J. Gen. Microbiol..

[39] Liu T., Chen Y.C., Jeng S.L., Chang J.J., Wang J.Y., Lin C.H., Tsai P.F., Ko N.Y., Ko W.C., Wang J.L. (2023). Short-term effects of Chlorhexidine mouthwash and Listerine on oral microbiome in hospitalized patients. Front. Cell Infect. Microbiol..

[40] Do T., Devine D., Marsh P.D. (2013). Oral biofilms: Molecular analysis, challenges, and future prospects in dental diagnostics. Clin. Cosmet. Investig. Dent..

[41] Bucki R., Namiot D.B., Namiot Z., Savage P.B., Janmey P.A. (2008). Salivary mucins inhibit antibacterial activity of the cathelicidin-derived LL-37 peptide but not the cationic steroid CSA-13. J. Antimicrob. Chemother..

[42] Abouassi T., Hannig C., Mahncke K., Karygianni L., Wolkewitz M., Hellwig E., Al-Ahmad A. (2014). Does human saliva decrease the antimicrobial activity of chlorhexidine against oral bacteria?. BMC Res. Notes.

[43] Spijkervet F.K., van Saene J.J., van Saene H.K., Panders A.K., Vermey A., Fidler V. (1990). Chlorhexidine inactivation by saliva. Oral. Surg. Oral. Med. Oral. Pathol..

[44] Ustrell-Borràs M., Traboulsi-Garet B., Gay-Escoda C. (2020). Alcohol-based mouthwash as a risk factor of oral cancer: A systematic review. Med. Oral. Patol. Oral. Cir. Bucal.

[45] Tokajuk J., Deptuła P., Chmielewska S.J., Skłodowski K., Mierzejewska Ż., Grądzka-Dahlke M., Tołstoj A., Daniluk T., Paprocka P., Savage P.B. (2022). Ceragenin CSA-44 as a Means to Control the Formation of the Biofilm on the Surface of Tooth and Composite Fillings. Pathogens.

[46] Niemirowicz K., Durnaś B., Tokajuk G., Piktel E., Michalak G., Gu X., Kułakowska A., Savage P.B., Bucki R. (2017). Formulation and candidacidal activity of magnetic nanoparticles coated with cathelicidin LL-37 and ceragenin CSA-13. Sci. Rep..

[47] Brooks S.E., Walczak M.A., Hameed R., Coonan P. (2002). Chlorhexidine resistance in antibiotic-resistant bacteria isolated from the surfaces of dispensers of soap containing chlorhexidine. Infect. Control Hosp. Epidemiol..

[48] Bhardwaj P., Ziegler E., Palmer K.L. (2016). Chlorhexidine Induces VanA-Type Vancomycin Resistance Genes in Enterococci. Antimicrob. Agents Chemother..

[49] Hashemi M.M., Holden B.S., Coburn J., Taylor M.F., Weber S., Hilton B., Zaugg A.L., McEwan C., Carson R., Andersen J.L. (2019). Proteomic Analysis of Resistance of Gram-Negative Bacteria to Chlorhexidine and Impacts on Susceptibility to Colistin, Antimicrobial Peptides, and Ceragenins. Front. Microbiol..

[50] Noto M.J., Domenico H.J., Byrne D.W., Talbot T., Rice T.W., Bernard G.R., Wheeler A.P. (2015). Chlorhexidine bathing and health care-associated infections: A randomized clinical trial. JAMA.

[51] Spałek J., Daniluk T., Godlewski A., Deptuła P., Wnorowska U., Ziembicka D., Cieśluk M., Fiedoruk K., Ciborowski M., Krętowski A. (2021). Assessment of Ceragenins in Prevention of Damage to Voice Prostheses Caused by. Pathogens.

[52] Pollard J.E., Snarr J., Chaudhary V., Jennings J.D., Shaw H., Christiansen B., Wright J., Jia W., Bishop R.E., Savage P.B. (2012). In vitro evaluation of the potential for resistance development to ceragenin CSA-13. J. Antimicrob. Chemother..

[53] McCullough M.J., Farah C.S. (2008). The role of alcohol in oral carcinogenesis with particular reference to alcohol-containing mouthwashes. Aust. Dent. J..

[54] Friedrich R.E., Kristen U. (2003). Toxicity assessment of mouthwashes in the pollen tube growth test. Anticancer. Res..

[55] Hostiuc S., Ionescu I.V., Drima E. (2021). Mouthwash Use and the Risk of Oral, Pharyngeal, and Laryngeal Cancer. A Meta-Analysis. Int. J. Environ. Res. Public Health.

[56] Lanzetti J., Finotti F., Savarino M., Gassino G., Dell’Acqua A., Erovigni F.M. (2023). Management of Oral Hygiene in Head-Neck Cancer Patients Undergoing Oncological Surgery and Radiotherapy: A Systematic Review. Dent. J..

[57] Thornton C.P., Li M., Budhathoki C., Yeh C.H., Ruble K. (2022). Anti-inflammatory mouthwashes for the prevention of oral mucositis in cancer therapy: An integrative review and meta-analysis. Support. Care Cancer.

[58] Colella G., Boschetti C.E., Vitagliano R., Colella C., Jiao L., King-Smith N., Li C., Nuoh Lau Y., Lai Z., Mohammed A.I. (2023). Interventions for the Prevention of Oral Mucositis in Patients Receiving Cancer Treatment: Evidence from Randomised Controlled Trials. Curr. Oncol..

[59] Rajendiran M., Trivedi H.M., Chen D., Gajendrareddy P., Chen L. (2021). Recent Development of Active Ingredients in Mouthwashes and Toothpastes for Periodontal Diseases. Molecules.

[60] Bucki R., Niemirowicz K., Wnorowska U., Byfield F.J., Piktel E., Wątek M., Janmey P.A., Savage P.B. (2015). Bactericidal activity of ceragenin CSA-13 in cell culture and an animal model of peritoneal infection. Antimicrob. Agents Chemother..

[61] Wnorowska U., Piktel E., Deptuła P., Wollny T., Król G., Głuszek K., Durnaś B., Pogoda K., Savage P.B., Bucki R. (2022). Ceragenin CSA-13 displays high antibacterial efficiency in a mouse model of urinary tract infection. Sci. Rep..

[62] Yousefimanesh H., Amin M., Robati M., Goodarzi H., Otoufi M. (2015). Comparison of the Antibacterial Properties of Three Mouthwashes Containing Chlorhexidine Against Oral Microbial Plaques: An in vitro Study. Jundishapur J. Microbiol..

[63] Masadeh M.M., Gharaibeh S.F., Alzoubi K.H., Al-Azzam S.I., Obeidat W.M. (2013). Antimicrobial activity of common mouthwash solutions on multidrug-resistance bacterial biofilms. J. Clin. Med. Res..

[64] Takenaka S., Sotozono M., Ohkura N., Noiri Y. (2022). Evidence on the Use of Mouthwash for the Control of Supragingival Biofilm and Its Potential Adverse Effects. Antibiotics.

[65] Zaugg A., Sherren E., Yi R., Larsen T., Dyck B., Stump S., Pauga F., Linder A., Takara M., Gardner E. (2023). Incorporating Ceragenins into Coatings Protects Peripherally Inserted Central Catheter Lines against Pathogen Colonization for Multiple Weeks. Int. J. Mol. Sci..

[66] Yazicioglu O., Ucuncu M.K., Guven K. (2023). Ingredients in Commercially Available Mouthwashes: A Review. Int. Dent. J..

[67] Ding B., Guan Q., Walsh J.P., Boswell J.S., Winter T.W., Winter E.S., Boyd S.S., Li C., Savage P.B. (2002). Correlation of the antibacterial activities of cationic peptide antibiotics and cationic steroid antibiotics. J. Med. Chem..

[68] Pfaller M.A., Sheehan D.J., Rex J.H. (2004). Determination of fungicidal activities against yeasts and molds: Lessons learned from bactericidal testing and the need for standardization. Clin. Microbiol. Rev..

[69] Sandberg M.E., Schellmann D., Brunhofer G., Erker T., Busygin I., Leino R., Vuorela P.M., Fallarero A. (2009). Pros and cons of using resazurin staining for quantification of viable Staphylococcus aureus biofilms in a screening assay. J. Microbiol. Methods.

[70] Dalecki A.G., Crawford C.L., Wolschendorf F. (2016). Targeting Biofilm Associated Staphylococcus aureus Using Resazurin Based Drug-susceptibility Assay. J. Vis. Exp..

[71] Greco I., Molchanova N., Holmedal E., Jenssen H., Hummel B.D., Watts J.L., Håkansson J., Hansen P.R., Svenson J. (2020). Correlation between hemolytic activity, cytotoxicity and systemic in vivo toxicity of synthetic antimicrobial peptides. Sci. Rep..

[72] Vishnepolsky B., Zaalishvili G., Karapetian M., Nasrashvili T., Kuljanishvili N., Gabrielian A., Rosenthal A., Hurt D.E., Tartakovsky M., Grigolava M. (2019). De Novo Design and In Vitro Testing of Antimicrobial Peptides against Gram-Negative Bacteria. Pharmaceuticals.

[73] Verma U.P., Gupta A., Yadav R.K., Tiwari R., Sharma R., Balapure A.K. (2018). Cytotoxicity of chlorhexidine and neem extract on cultured human gingival fibroblasts through fluorescence-activated cell sorting analysis: An. Eur. J. Dent..

